# Free Tissue Transfer in the Reconstruction of Neck Contractures after Burn Injury: A Case Series

**DOI:** 10.3390/ebj4020022

**Published:** 2023-06-09

**Authors:** Geneviève Ferland-Caron, Peter O. Kwan, Edward E. Tredget

**Affiliations:** 1 Department of Surgery, University of Montreal, Montreal, QC H1Y 3L1, Canada; 2 Wound Healing Research Group, Division of Plastic and Reconstructive Surgery, University of Alberta, Edmonton, AB T6G, Canada; 3 Firefighters’ Burn Treatment Unit, Division of Plastic Surgery, Department of Surgery, University of Alberta, Edmonton, AB T6G, Canada

**Keywords:** burn scar contracture, free tissue transfer, microsurgery, scars

## Abstract

**Background**: Recent advances in burn care have significantly improved the survival rate of patients with extensive burn injuries, placing greater emphasis on reconstruction to improve the long-term outcomes of scar deformities. Anterior and lateral neck contractures are common after burn injuries; they limit range of motion, complicate airway management and create significant cosmetic deformities. Traditional methods have been used to release contractures and improve function. However, they are subject to variable results, residual neck tightness, recurrence and suboptimal cosmetic appearance. Microvascular free tissue transfer is a more technically challenging and time-consuming method, but it offers the potential to overcome the long-term limitations of simpler options. In this paper, we present our experience with microvascular free flaps for the release of burn scar contractures of the neck as a potential high-quality permanent solution. **Methods**: Over a 10-year period, nine free flaps were performed on burn patients with total body surface area (TBSA) burns between 20 and 70%, who developed moderate to severe neck contractures. Four anterolateral thigh (ALT) flaps, four radial forearm free flaps (RFFFs) and one ulnar forearm flap were used to release neck contractures. **Results**: All nine flaps were completed successfully with significant improvement in the neck’s range of motion. Good aesthetic results were achieved with smooth contour and thin coverage. Overall, the patients were satisfied. However, five out of nine cases required at least one secondary procedure for flap defatting to reach optimal results. **Conclusion**: Post-burn scar contractures of the cervical region compromise the cosmetic appearance and airway security of recovering burn patients, imposing a significant impact on their psychological and functional quality of life. Consequently, cervical contractures can be prioritized when planning reconstruction for burn patients. Free flaps can be considered an important and reliable method of reconstruction for neck contracture deformity following burn injuries.

## 1. Introduction

Improvements in burn care over the 20th century have not only decreased the mortality rate but have also increased attention towards a reduction in morbidity and innovative techniques to improve quality of life. The head and neck area has a high prevalence for burns [1]. The neck is also an anatomic zone with multidirectional function, and scar contractures have tendency to form easily, mostly in a flexed position secondary to the gravity effect of the head in standing position [2]. Neck contractures can affect other functional areas by putting excessive tension on facial soft tissue, affecting expression, eye protection, lip closure and airway access. Distorted appearance may create psychological problems and decrease confidence for social interaction.

The reconstruction of a neck contracture post-burn injury is challenging for many reasons, partly because of the insufficient tissue available to resurface large defects, but also it requires high-quality tissue to achieve a satisfying cosmetic appearance.

Multiple methods have been applied when a contracture is limited to a small area including partial and full-thickness skin grafts, Z-plasties, local flaps, tissue expanders, skin substitutes and lasers [3]. These techniques are less time-consuming, technically easier to perform and offer quicker recovery. However, their disadvantages are primarily limited to large burns where recurrence remains frequent and success with grafted materials is less reliable. Moreover, when the contractures are extensive, the adjacent area is burned and large donor areas are available, microsurgical reconstruction of the neck has been shown to offer better functional results and reduce morbidity [4,5].

The objectives of neck contracture reconstruction should include cosmetic outcome, symmetry, contour, color and texture match and should also restore mobility, preventing excessive pull on facial soft tissues [5]. Furthermore, early reconstruction can minimize the risk of a difficult airway for further surgical intervention [6].

Microsurgery has been used in burn reconstruction since 1960 [7]; however, microsurgical procedures on burn patients are especially challenging, and they not only need greater flexibility in the selection of the flap but are also more time-consuming. Realizing a free flap in a burn patient needs more imaginative planning and can be more complicated because of many factors such as venous damage, zone of trauma affecting the quality of the recipient vessels and lack of donor sites due to the burn itself or the harvesting of a skin graft [8].

In this article, we present a series of nine patients with reconstruction of neck contractures by free flaps.

## 2. Patients and Methods

The authors reviewed cases of neck burn reconstruction performed at the University of Alberta Hospital, Canada, over a 10-year period from 2005 to 2016. A total of 9 patients with extensive neck contractures underwent free-flap reconstruction at this institution; 5 were male and 4 were female, their ages ranging from 5 to 59 years old (mean 31.7 years). The etiologies of the neck burns were flame in 7 patients, scald burns in one patient and electrical burns in one patient. The indication for free flap for these patients was limitation in neck movement affecting quality of life, in spite of aggressive rehabilitation programs, with or without previous surgical release. The choice of free flaps used included 2 radial forearm flaps (RFF), 2 radial forearm pre-expanded flaps, 4 anterolateral thigh flaps (ALT) and one ulnar forearm flap. The flaps were used to resurface the neck area after removal of the scar tissue causing the neck contractures.

Statistical analyses were performed for the four more-recent patients to determine the effect of reconstruction on the ROM. Analysis was performed with two-tail paired *t* test. The statistical significance was considered with a *p* value ≤ 0.05 and was obtained for those four patients. The neck range of motion was measured using specific cervical movements such as bilateral side flexion, extension and bilateral lateral rotation. Physiotherapists of the burn unit conducted these measurements preoperatively and then at 1 and 3 months post-operatively.

### Surgical Technique

A two-team approach was used to prepare the recipient vessels and harvest the flap at the same time. The first team worked at the neck and completely released the contracture, and resection of the platysma muscle was required when present in some cases. Following the release and resection, the first team assessed the size of the defect to determine the canvas for the flap. They continued with the exposure of the recipient vessels; these consisted of the facial artery and vein in 8 of the 9 cases and the ascending pharyngeal artery and internal jugular vein in one case. In some cases, the submaxillary gland was resected to improve access to the facial artery and vein and to create space for the pedicle of the flap after wound closure. The second team proceeded with the harvesting of the flap and closure of the donor site, which was accomplished primarily in two patients and with a split thickness skin graft in the others. After completion of recipient vessel exposure and flap harvesting, surgical anastomoses of one artery and vein were performed with microsurgical techniques using coupling devices on the venous anastomosis. Flap monitoring was assisted by implantable doppler devices around the arterial pedicle and venous monitoring visually by experienced nurses in the burn intensive care unit.

## 3. Case Reports

### 3.1. Case 1

**Figure 1:**
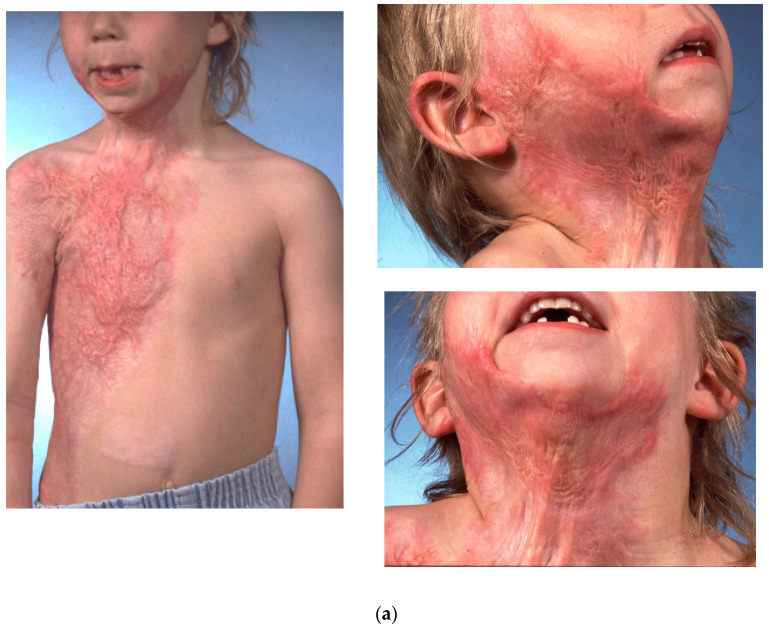

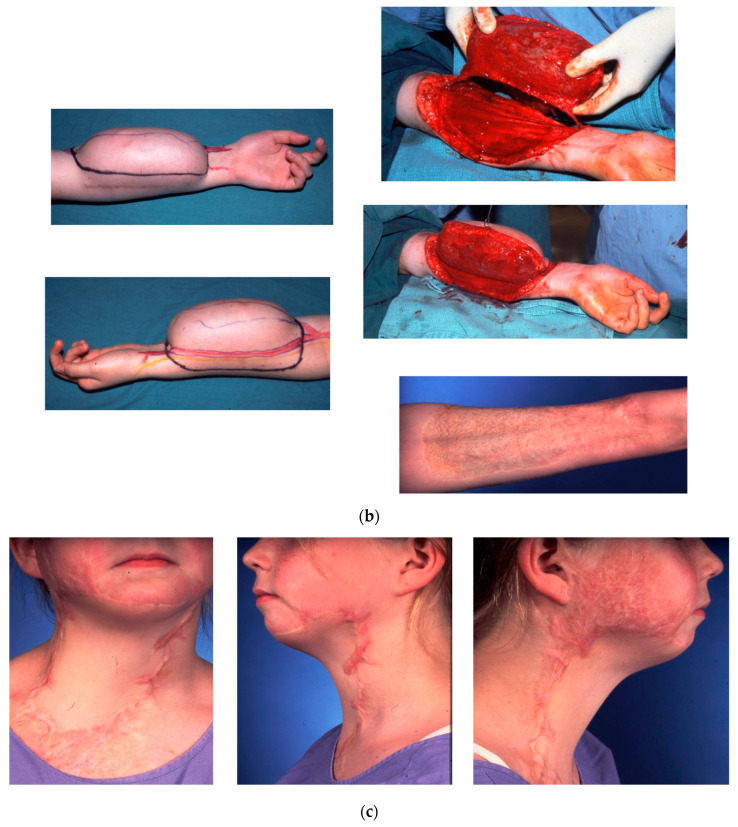
A five-year-old child who suffered 35% TBSA burn involving the head and neck causing significant hypertrophic scar and neck contracture (**a**). Images depict the appearance of the fully expanded soft tissue expander prior to free tissue transfer using the pedicle of the radial artery supplying the flap. The bottom right figure illustrates the skin grafted and healed donor site 2 months after surgery (**b**). The post-operative result is illustrated at 2 months after the completed surgery (**c**).

This patient was a five-year-old girl injured over 8% TBSA from a hot water scald who developed hypertrophic scars of the face, chest and right arm in the first year after the injury (Figure 1a).

She underwent right radial forearm free-flap coverage after placement of a subcutaneous soft tissue expander and progressive expansion over 4 months. The expander was a 100 cc total volume rectangular expander with a self-contained port placed under the forearm fascia via an ulnar-based incision. Appearances of the expanded tissue prior to free tissue transfer and the resultant skin grafted donor site are shown in Figure 1b.

Her immediate post-operative appearance 2 months after reconstruction before further treatment was conducted of the hypertrophic scars at the border of the flap.

### 3.2. Case 2

**Figure 2:**
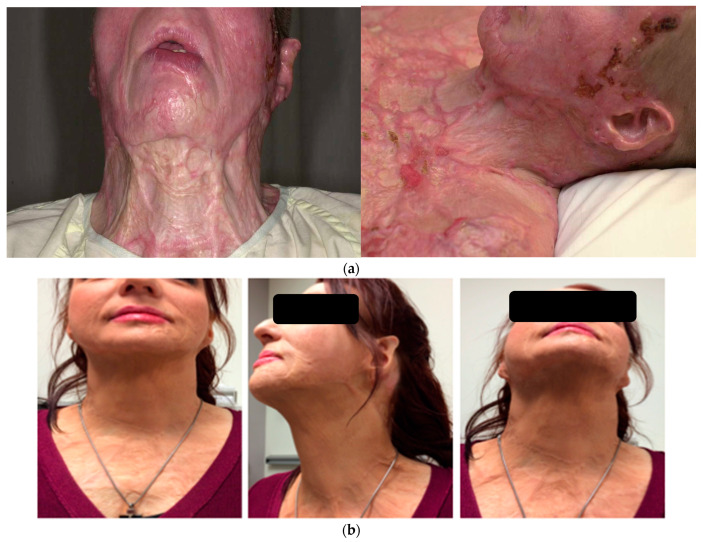
(**a**) Scar appearance 3 months post-burn injury. She underwent right anterior lateral free-flap coverage of the neck after scar release. Shown is the post-operative appearance of the neck (**b**) 5 years after surgery and two defatting procedures.

A 34-year-old woman was injured over 40% TBSA in a house fire and reconstructed with split thickness skin grafts. She developed severe neck contractures with disfiguring burn scars (Figure 2a), as seen 3 months post-burn injury. She underwent right anterior lateral free-flap coverage of the neck after scar release. Shown is the post-operative appearance of the neck (Figure 2b) and donor site (Figure 2c), 5 years after surgery and two defatting procedures.

### 3.3. Case 3

**Figure 3:**
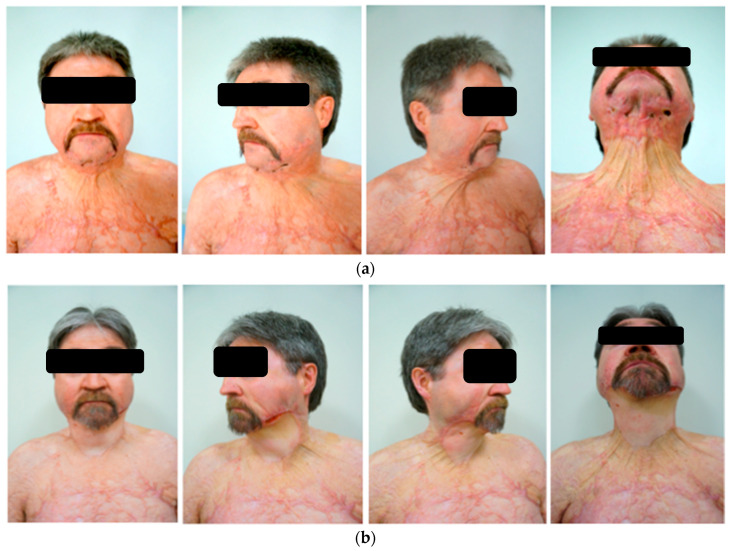
(**a**) A 57-year-old man with burn scar contractures of the neck prior to surgery, illustrating the four ranges of motion measured in the study. (**b**) Appearance 6 months post-operatively, after one defatting procedure.

A 57-year-old man sustained flame burn to 43% of his body involving the anterior neck, face, upper extremities and chest, one year before reconstructive surgery. Conservative treatment and local flap had left an extensive contracture and hair inclusions creating hygiene issues and recurring folliculitis unresponsive to medical treatment (Figure 3a). He underwent scar release and reconstruction of the neck with a free anterolateral thigh flap. First, the contracture in the neck was released and an ALT flap was outlined. Microsurgical anastomosis of the flap to the facial artery and vein was performed. Once the viability of the flap was confirmed, the neck scar was resected in a full-thickness excision. The flap donor site was closed with a split-thickness skin graft. Two months post-operatively, a defatting procedure was performed with direct excision and suction lipectomy. This secondary procedure was complicated by necrosis of the caudal edge of the flap. After conservative treatment, the patient was satisfied and left with a natural-looking profile and satisfying cosmetic results. Long-term follow-up at 6 months demonstrated an improvement in appearance, hygiene and range of motion (Figure 3b).

## 4. Results

Free tissue transfers have been shown to be reliable for neck contractures [8] and result in an improvement in appearance with pliable tissue. The contracture release was successful in all nine patients included in the study. Overall, nine of the nine flaps survived and healed well, and no re-exploration was required. The patients were monitored in the burn intensive care unit, and an implanted arterial Doppler probe was used in five patients.

A debulking procedure was offered to patients if the flap remained bulky for more than two months after the intervention. Fat was then removed from the underside of approximately half of the flap, and a suction lipectomy was used to increase the contouring effect. Shaping the flap to obtain good contouring is the key to a good outcome. One debulking procedure was complicated by necrosis of the caudal edge. Six flaps required secondary surgery to improve the contour, and three flaps required Z-plasty to release contractures on the incision site. Lasers were also used to remove any undesirable hair.

In this series, we demonstrated one of the main advantages of free-flap reconstruction for neck contracture: the durability of reconstruction. The patients were able to achieve a good range of motion (ROM) and maintain it for the duration of follow-up. Even with the absence of rehabilitation programs, patients who were followed for a longer period exhibited no recurrence of contracture. Cervical range of motion was significantly improved with free-flap reconstruction. The improvement in function was based on the measurements of neck movements.

**Figure 4:**
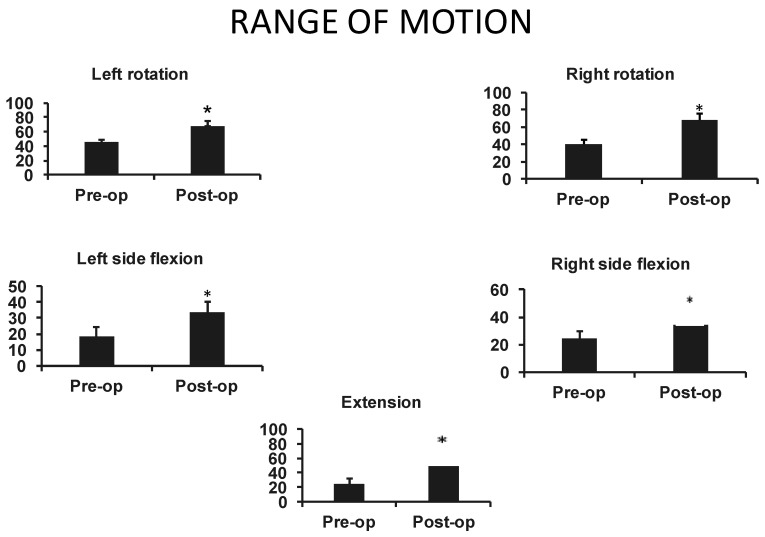
Neck range of motion prior to surgery and after free-flap reconstruction. * *p* < 0.05.

As illustrated in Figure 4, both right and left rotation and flexion were significantly increased in the post-operative period as well as neck extension.

In our series, most of our patients were classified as difficult intubation where the Mallampati score was greater than 3, due to a mentosternal distance less than 12.5 cm and inter-incisor gap less than 4 cm. However, after neck release and free-flap coverage, there was an improvement in airway access, making further reconstructive surgery easier and more secure.

In our study, improvement in ROM was achieved within a short period of time.

Post-operatively, the physiotherapy team proceeded with the teaching of gentle exercises starting 1 week after surgery and implemented a home maintenance program to reduce neck stiffness. Patients were seen in physiotherapy at 1 week, 1 month and 3 months for follow-ups. Despite no intensive rehabilitation program and no uncomfortable pressure garments or splints, recurrence of the contracture during the long-term follow-up was not observed.

Long-term follow-up pictures show the resulting cosmetic improvement, contracture release, contour and color match. Patients found that imperfect color matches were easily concealable with minimal make-up because the smoothness and texture of the flap skin resembled normal neck skin in the region.

### Complications

Minor complications were observed, and they included: donor site rash and granulation tissue, as well as necrotic flap edges after debulking. One patient suffered a methicillin-resistant *Staphylococcus aureus* (MRSA) infection of the donor site expander, which was removed and treated with debridement and intravenous Vancomycin. After several months, he returned for surgical release and reconstruction with an unexpanded radial forearm flap from the same donor area.

## 5. Discussion

The goal of neck contracture reconstruction is to recreate a natural-looking profile but also protect from recurrences, seen frequently with traditional methods and necessitating religious use of a splint device. Moreover, the cervical area requires high flexibility; this can be difficult to achieve with a skin graft because of primary and secondary contractures. Adequate contour and color are also difficult to achieve with grafting [5]. In addition, Desmouliere et al. have demonstrated that flap coverage of the open wound inhibits wound contracture and hypertrophy likely through the induction of apoptosis in the underlying fibroblasts and myofibroblasts and enhanced remodeling of granulation tissue in the wound bed [9]. This may be a biologic explanation for the improvement in wound healing, reduction in scar contracture and control of scar hypertrophy with flaps as compared to skin grafts.

Pedicle and local flaps such as supraclavicular-artery-based or transverse cervical-artery-based flaps can allow good coverage and long-term release of the contractures; however, local flaps on burn patients are typically restricted by the extent of the injury zone [8,10]. Moreover, the additional scars from loco-regional donor sites, mostly on the shoulders or chest, may not be considered acceptable by patients [5]. Soft tissue expanders can offer an attractive option [11], but the gain obtained is rarely sufficient for resurfacing an extensive neck contracture, and significantly more clinic visits and time are required to complete the expansion. Moreover, stretching created by this technique often decreases with time, increasing the rate of contracture recurrence [11]. Therefore, only a few patients with mild neck contractures can be treated by these methods.

Free flaps offer wide coverage, a low rate of contracture recurrence and a natural-looking contour with a cosmetically satisfying appearance [8,10]. As a disadvantage, the use of free flaps leaves a scar at the donor site that can be significant depending on the size of the flap required and the type of flap chosen.

Different free tissue transfers have been described for the correction of cervical burn contractures. These consist mostly of fasciocutaneous flaps such as radial forearm, groin flaps [12], anterolateral thigh flaps [13,14], scapular flaps and thoracodorsal artery perforator flaps [15]. As discussed by Duteille et al., fasciocutaneous flaps from the anterior part of the body, as described in this series, are preferable because of their pliability, and they facilitate better positioning for the two-team approach [16]. Some authors also described the advantages of a pre-expanded free flap, as this allows the flap to be made thinner and larger while minimizing the donor site morbidity [5,17]. In our series, we used the expansion for three of the radial forearm flaps, but we were successful only in two due to infectious complications in healing of the expander after insertion.

**Figure 5:**
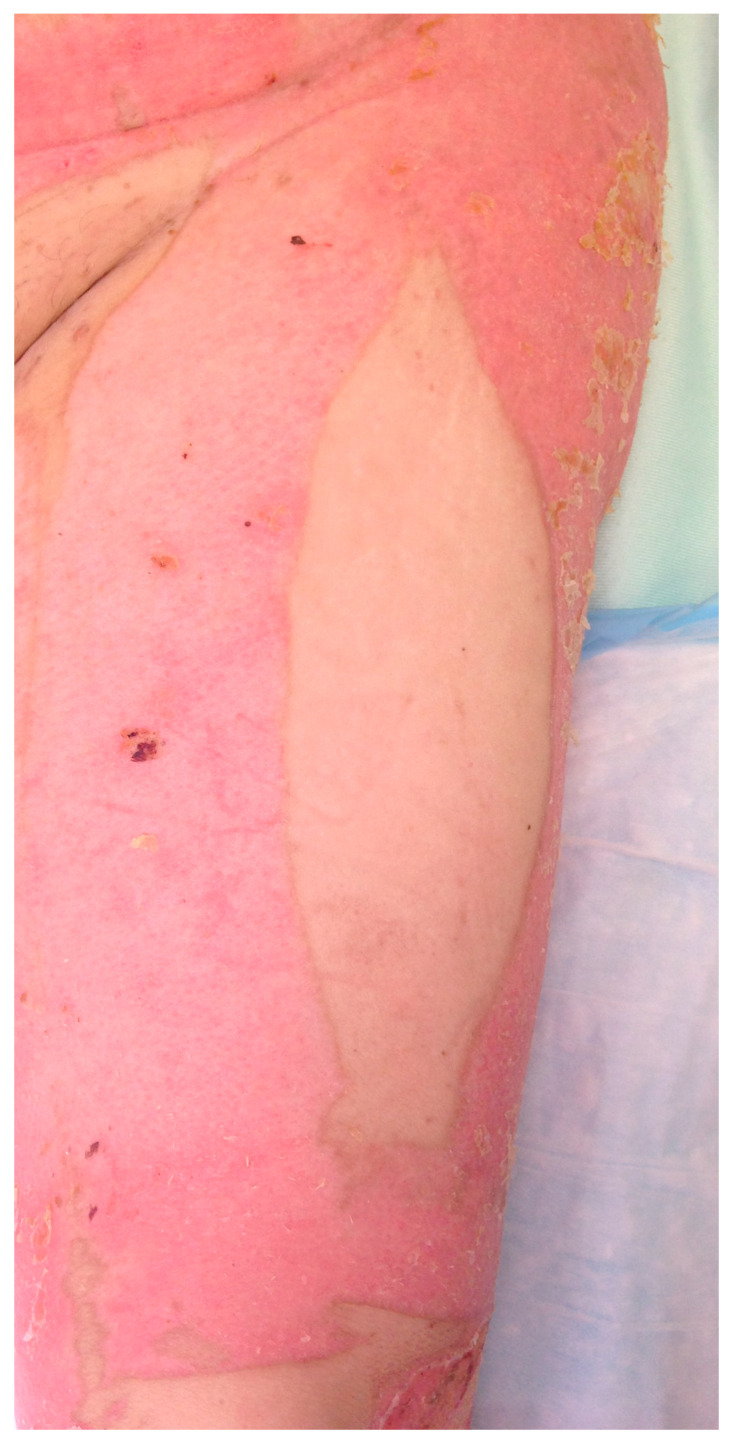
An ALT donor site on the left thigh was not harvested for skin grafts so that it could be conserved for future reconstruction of the neck in the burn patient.

As a result of this series, our group has adopted a strategy similar to Tsai et al. [2], where the thigh is considered an ideal donor site with minimal donor site morbidity [18]. Therefore, when treating major burn of the head and neck, we spare this region from skin harvesting, if possible, in order to preserve it for eventual reconstructive needs [2] (Figure 5).

### Airway Management

Securing the airway in delayed neck reconstruction is crucial and can be challenging because of anatomical variations and the lack of cervical motion. A portion of burn patients with neck contractures also presented airway involvement at the initial injury, which influenced airway management [6]. The limitation of cervical range of motion, perinasal or circumoral scarring, decreased mouth opening, distortion of the larynx and the mandible or a history of inhalation injury need to be assessed by an experienced anesthesiology team. Vigilance, judicious pre-op assessment and teamwork are the keys to success. The potential for difficult intubation is predicted by a Mallampati score greater than 3 and includes a mentosternal distance of less than 12.5 cm and an inter-incisor gap of less than 4 cm [19,20]. A surgeon should always be ready during the intubation process for possible scar release and tracheotomy. Surgical scar release prior to intubation under general anesthesia has been described; however, in experienced centers it is mostly preferred to proceed with intubation first using different tools such as a GlideScope^®^ video laryngoscopy and awake blind or fiberoptic-assisted bronchoscopy. Intra-operatively, after successful intubation, the endotracheal tube was secured during the procedure using interdental wiring with 24-gauge stainless-steel wire in an Ivy wire loop fashion, as we have previously described [19]. This approach afforded secure fixation of the tube throughout the procedure despite movement of the head and neck and unobstructed access to the skin of the lower facial and neck region. Cosmetic reasons alone already place cervicofacial contractures at a priority for reconstruction, but surgeons should also prioritize this region in order to decrease the risk associated with difficult airway access for further reconstructive procedures [20].

Within our series, we have demonstrated an improvement in airway access based on an improvement in their Mallampati scores. Some patients went from difficult awake intubation requiring awake fiber optic intubation to regular Glidescope intubation, which we consider a significant improvement for the safety and discomfort of further reconstructive and emergency procedures.

## 6. Conclusions

In conclusion, in our experience, free flaps have developed as a reliable permanent option for cervical contracture release. Our patients had improved range of motion and appearance shortly after the surgery, with no major complications, and early neck reconstruction can improve the airway access for further surgical or emergency interventions.

Although our study demonstrates similar results with ALT and RFF, the donor site for the RFF is a distinct disadvantage of this flap. Even if several stages of revision may be needed to remove the excess bulk, the ALT flap remains our flap of choice for neck reconstruction. Attention must be paid to insetting of the flap and secondary defatting procedures to recreate a smooth contour [14,18]. Optimal results are obtained by minimizing the donor site morbidity while scar resurfacing is achieved, which can be challenging in the neck area mainly because of the large size of the area involved and the damage to free-flap donor tissue after major burn injury. In our experience, microsurgery has become a safe and reliable method for burn reconstruction and can achieve superior results in function and cosmetic appearance.

### Limitations of the Study

The number of patients in our study is small, and the subset of patients who were studied in a prospective fashion was limited. Patients described in the study included individuals who were first operated on more than twenty years ago, which made follow up difficult. Larger numbers of cases in a controlled trial with further measurements of range of motion, color matching and Mallampati scores would improve the strength of the findings of this case series of patients.

## Data Availability

Data are available by request using the email etredget@ualberta.ca.
